# Direct imaging of local atomic structures in zeolite using optimum bright-field scanning transmission electron microscopy

**DOI:** 10.1126/sciadv.adf6865

**Published:** 2023-08-02

**Authors:** Kousuke Ooe, Takehito Seki, Kaname Yoshida, Yuji Kohno, Yuichi Ikuhara, Naoya Shibata

**Affiliations:** ^1^ Institute of Engineering Innovation, School of Engineering, the University of Tokyo, Yayoi 2-11-16, Bunkyo, Tokyo 113-0032, Japan.; ^2^ Nanostructures Research Laboratory, Japan Fine Ceramics Center, Mutsuno 2-4-1, Atsuta, Nagoya 456-8587, Japan.; ^3^ PRESTO, Japan Science and Technology Agency, Kawaguchi, Saitama 332-0012, Japan.; ^4^ JEOL Ltd., 1-2-3 Musashino, Akishima, Tokyo 196-8558, Japan.

## Abstract

Zeolites are used in industries as catalysts, ion exchangers, and molecular sieves because of their unique porous atomic structures. However, direct observation of zeolitic local atomic structures via electron microscopy is difficult owing to low electron irradiation resistance. Subsequently, their fundamental structure-property relationships remain unclear. A low-electron-dose imaging technique, optimum bright-field scanning transmission electron microscopy (OBF STEM), has recently been developed. It reconstructs images with a high signal-to-noise ratio and a dose efficiency approximately two orders of magnitude higher than that of conventional methods. Here, we performed low-dose atomic-resolution OBF STEM observations of two types of zeolite, effectively visualizing all atomic sites in their frameworks. In addition, we visualized the complex local atomic structure of the twin boundaries in a faujasite (FAU)–type zeolite and Na^+^ ions with low occupancy in eight-membered rings in a Na-Linde Type A (LTA) zeolite. The results of this study facilitate the characterization of local atomic structures in many electron beam–sensitive materials.

## Teaser

Optimum bright-field electron microscopy unveils atomic structures of zeolite, a popular yet beam-sensitive porous material.

## INTRODUCTION

Zeolites are porous materials with regularly arranged nanosized pores, which enable a wide range of applications in catalysis, gas separation, and ion exchange (*1*, *2*). The material properties of zeolites are closely related to the geometry of their pores and their subsequent interactions with any adsorbed guest molecules and ions. To date, diffractometric techniques have been most often used for the structural analysis of zeolites (*3*). Although diffraction methods can accurately analyze averaged structures, obtaining local structural information related to defects, interfaces, and surfaces is extremely difficult. Scanning transmission electron microscopy (STEM) is a powerful technique for local structural analysis that enables the direct observation of atomic structures in electron-resistant materials at a subangstrom resolution (*4*). However, zeolites are more electron beam–sensitive than other inorganic materials. Thus, atomic-scale observations via electron microscopy are severely limited by electron irradiation damage (*5*, *6*). Menter (*7*) observed faujasite (FAU) zeolite in 1958 via high-resolution transmission electron microscopy (HRTEM) and reported a lattice resolution of 14 Å. Subsequently, the zeolite framework structure was observed (*8*). In the 1990s, an aberration corrector was developed, and the S/TEM resolution was substantially improved (*9*). These technological advances have enabled the direct observation of the framework structure and arrangement of adsorbed cations in zeolites (*10*). However, it remains extremely challenging to directly observe all the atomic sites in zeolites, including the Si/Al and oxygen sites, owing to the severe electron irradiation damage within zeolites.

**Fig. 1.**
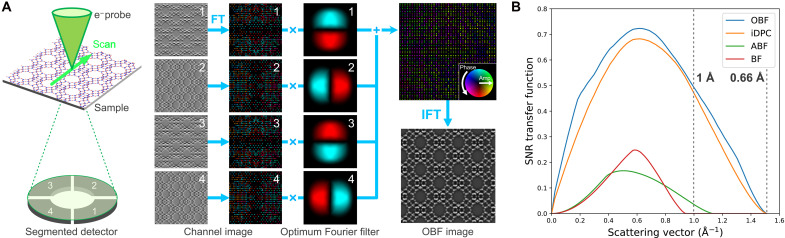
Reconstruction scheme of OBF STEM and dose-efficiency comparison based on SNR transfer functions for different STEM imaging techniques. (**A**) Schematic illustration of OBF STEM image processing workflow. In OBF STEM, a segmented detector is located on the diffraction plane that collects the intensity of transmitted/diffracted electrons at each probe position. The STEM images acquired by each segment are then processed with frequency filters to extract the phase-contrast component. The frequency filters are derived via STEM CTF, which are of a complex value. Subsequently, the filters are also complex-valued and visualized as a color map representing the phase and amplitude. After filtering, all the images are summed, and the OBF image is synthesized. As the filter is calculated via microscope optical information such as accelerating voltage and convergence angle of the probe as well as the CTF, OBF reconstruction does not need a priori knowledge of the sample. (**B**) SNR transfer functions of OBF and various phase-contrast imaging techniques. CTFs show the window of contrast transfer from samples as a function of spatial frequency. SNR transfer function is calculated by normalizing CTFs based on the noise level at each spatial frequency within the Poisson statistics, which shows a proportionality factor for the sample potential and electron dose to determine the SNR at each Fourier component. Here, the SNR transfer functions are calculated at an accelerating voltage of 300 kV, a convergence semi-angle of 15 mrad, and a sample thickness of 10 nm, the same conditions as those of the experiments conducted in this study. These transfer functions are shown as radially averaged values, and the OBF technique shows a higher SNR transfer than both the conventional methods (BF and ABF) and iDPC, the recently developed phase imaging technique.

Recent developments of STEM electron detectors have led to more advanced imaging techniques. Although conventional STEM uses a single annular detector to detect transmitted/scattered electrons to form images, the recently developed segmented/pixelated detectors can simultaneously form many STEM images using electrons detected in multiple areas on the diffraction plane. By processing these multiple STEM images, information regarding the electromagnetic fields and phase information of samples can be obtained (*11*, *12*). We have theoretically developed an optimum bright-field (OBF) STEM technique for low-dose imaging that enables the observation of atomic structures at the highest signal-to-noise ratio (SNR) using segmented/pixelated detectors (*13*). Figure 1A shows the schematic of the OBF STEM technique, which uses a segmented detector. Here, a finely focused electron probe is raster-scanned across the sample, and the transmitted/scattered electrons are detected at each raster by a multiple-segmented electron detector. Subsequently, frequency filters are applied to each image obtained by the corresponding detector segment, and the filtered images are assembled to obtain the OBF image. These frequency filters select the Fourier components where higher image contrast is transferred against the noise in each segment and are designed to theoretically maximize the SNR of the synthesized image based on the STEM contrast transfer function (CTF) (*14*) and noise-evaluation theory (*15*). Figure 1B presents the SNR transfer function for various phase-contrast STEM techniques. The SNR transfer function represents a proportionality factor for the sample potential and electron dose to determine the SNR at each spatial frequency, which is helpful in fairly evaluating the imaging efficiency of different techniques (*15*) (details are described in Supplementary Text). Therefore, the imaging technique with a higher SNR transfer function obtains better SNR under the same sample potential and dose condition. OBF STEM, which uses a segmented detector, exhibits a much higher imaging efficiency than those of conventional techniques such as annular bright-field (ABF) and conventional bright-field (BF) imaging (*16*) over an entire spatial frequency domain. In addition, OBF is more efficient than integrated differential phase-contrast (iDPC) imaging (*17*), which is a phase imaging technique that also uses a segmented detector. As described in the Supplementary Text, OBF imaging achieves a dose efficiency approximately two orders of magnitude higher than those of the conventional STEM imaging methods and is ~24% higher than that of iDPC imaging. While this method has been applied to atomic-scale analysis of zeolitic frameworks and its interaction with guest species (*18*–*20*), OBF should be more promising for obtaining better experimental images with less irradiation damage and/or higher spatial resolution owing to the better efficiency and visualization as discussed later. It is also noted that the SNR transfer functions shown in Fig. 1B are calculated for the segmented/conventional detectors. However, pixelated detectors are rapidly advanced, and phase imaging techniques using this type of detector (i.e., electron ptychography) are also applied to zeolite framework imaging (*21*). The OBF technique can also be further improved by pixelated detectors, and the comparison including the pixelated type is discussed in Supplementary Text and fig. S5. Furthermore, OBF can obtain information at much higher spatial frequencies (i.e., higher resolution) than those of the conventional techniques, as shown in Fig. 1B. In this study, we obtained atomic-level structural images of zeolite via aberration-corrected STEM with an accelerating voltage of 300 kV and a probe-forming aperture of 15 mrad. In OBF STEM, the information limit, a maximum spatial frequency of contrast transfer in the Fourier space, is given as 2*k*_0_ (*k*_0_ denotes a radius of the convergence aperture in the reciprocal space). Thus, OBF can collect the sample information with a frequency range of 0 to 2*k*_0_, where the maximum obtainable resolution is calculated to be 0.66 Å in real space. This indicates that the present optical condition is sufficient for obtaining atomic-resolution images.

Furthermore, the OBF images could be reconstructed in real time (*13*). In a typical atomic-resolution STEM operation, fine optical tuning adjustments, such as astigmatism correction, defocus correction, and field-of-view (FOV) adjustment, are performed by an operator who refers to atomic-resolution images displayed on a monitor in real time. In low-dose observations, fine-tuning becomes much more difficult because the operator cannot observe atomic structures in the real-time images owing to poor SNR. However, the photomultiplier-based segmented detector enables the dwell time of the electron probe to be as short as that of conventional detectors, and the synthesized images can also be processed as live imaging (*22*). Thus, by implementing a real-time OBF imaging function combined with a high-speed segmented detector and rapid scanning, an operator can observe atomic structures in real time with a higher SNR and tune the optical parameters even under low-dose conditions, as shown in movie S1. This technique facilitates the observation of beam-sensitive materials with minimal irradiation damage.

In this study, we used real-time OBF imaging to observe FAU-type zeolites at Si/Al = 50 with subangstrom resolution. Because this sample has open framework structure but rather low aluminum content, it is expected that it should be much more beam sensitive than the ordinary inorganic materials but not extremely weak compared with other zeolites with higher aluminum content. Therefore, this sample should be a suitable model system to evaluate the capability of low-dose OBF imaging technique. We demonstrated that OBF imaging allows the direct observation of the T (= Si, Al) and oxygen atoms in the TO_4_ tetrahedron building units, which constitute the FAU-type framework structure. Furthermore, OBF imaging was used to observe the detailed atomic structure of a twin boundary, which is a common lattice defect in FAU zeolites. Furthermore, we also applied this technique to Na-captured Linde Type A (LTA)-type zeolite at Si/Al = 1, known as one of the most beam-sensitive zeolite samples. The results of this study highlight the capability of electron microscopy for the local structural characterization of beam-sensitive materials.

## RESULTS

### Direct imaging of atomic structures in FAU zeolite

**Fig. 2.**
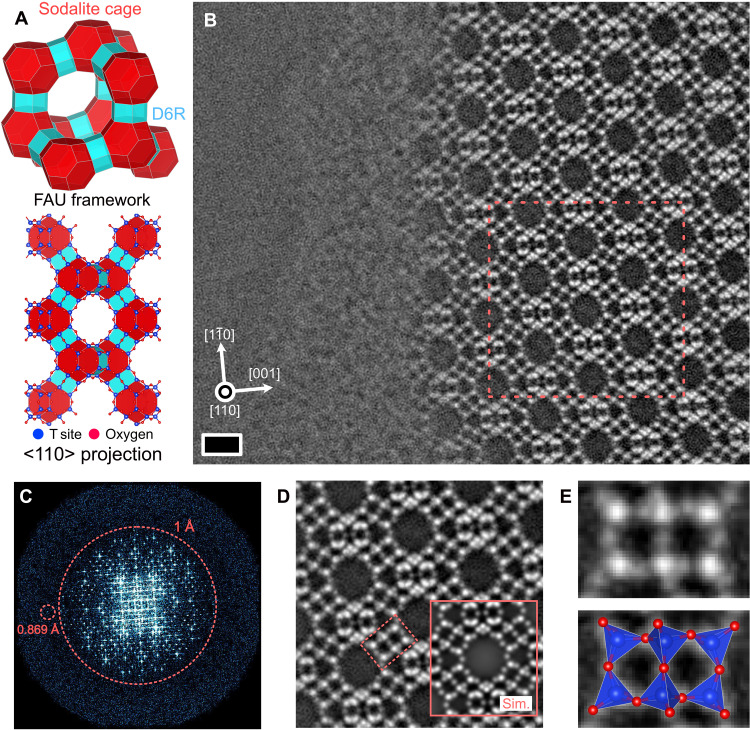
Atomic-resolution OBF STEM observation of an FAU zeolite along <110> zone axis. (**A**) Schematic of the FAU zeolite framework structure and projected atomic structure model along <110> zone axis. Red and blue polygons represent the building units (sodalite cages and D6Rs, respectively). (**B**) OBF STEM image of FAU zeolite observed at the edge of the sample. Bright spots indicate T and oxygen sites. Scale bar, 1 nm. The dashed rectangular indicates the repeat unit structure used for the averaging process shown in (D). (**C**) Fourier transform spectrum of (B), wherein the spots are seen up to 0.869 Å resolution in real space. (**D**) Repeat-unit-cell–averaged OBF image, which is obtained by cropping and averaging the multiple subimages obtained from the raw image shown in (B), offering a higher SNR. The inset is a simulated OBF image calculated with the same observation condition as that in the experiment. The location of the D6R structure, which is shown in (E), is highlighted by a dashed rectangular. (**E**) Magnified OBF image of the rectangular region indicated by the red dashed line in (D). The atomic structure models are drawn using visualization for electronic and structural analysis software (*49*).

Figure 2A schematically shows the FAU framework, which consists of two building blocks: sodalite cages (SODs) and double 6-membered rings (D6Rs). The lattice constant of FAU is *a* = *b* = *c* = 24.3450 Å and α = β = γ = 90° with a space group *Fd-*3*m*. In FAU, SODs are connected to the nearest four SODs through D6Rs as carbon atoms in diamond and form large pores of 12 Å in diameter. Implementing the real-time OBF imaging technique, we observed the FAU framework along the <110> zone axis, wherein the pores were aligned along the observation direction. Conventionally, the atomic structure from the same zone axis was observed via HRTEM (*23*), but only the pore arrangements were resolved by this method, and the atomic resolution was difficult to achieve. While this pore-arrangement resolution is sufficient to analyze the basic framework structures of zeolites as a set of building blocks, a higher spatial resolution is required for characterizing inhomogeneous structures such as lattice defects and extra-framework cations, which are analyzed here. Here, the electron probe current was set to 0.5 pA to suppress the beam damage, which was approximately two orders of magnitude lower than that of the usual STEM observation condition for analyzing typical inorganic materials. Under these conditions, OBF STEM observations of the FAU-type zeolite sample were conducted, as shown in Fig. 2 (B to E). Figure 2B shows the experimental OBF image of the FAU-type zeolite. The OBF image of the FAU framework structure indicated the atomic sites as bright spots, evidently for the tetrahedral (T sites occupied by Si or Al) and oxygen sites. An amorphous layer covering the sample surface is also recognizable in the image. In the literature of HRTEM study on FAU-type samples (*24*), while clean surface structures of FAU framework was observed in as-synthesized form, the surface-covering amorphous layer was confirmed after dealumination process to increase the Si/Al ratio, which is the same situation as the present study shown in Fig. 2B. Figure 2C shows the power spectrum of the OBF image in Fig. 2B that exhibits an information transfer up to 0.869 Å. Furthermore, Fig. 2D shows a unit cell–averaged OBF image obtained from the original OBF image in Fig. 2B. The FAU framework structure can be observed at an atomic resolution and conforms extremely well with the simulated image shown in the inset, indicating that the atomic structure can be resolved without any electron irradiation damage. Figure 2E is the cropped image of Fig. 2D, focusing on the D6R building block of the FAU structure. It evidently shows that the tetrahedral units are connected via corner-shared oxygen sites with a centrosymmetry. In zeolites, the oxygen-bridging sites between the tetrahedral units play an essential role in dictating the properties exhibited by the material, such as the catalytic activity (*25*) and structural transformation introduced by the interactions between the framework host and captured guests (*26*). Thus, the capability of OBF STEM to visualize individual oxygen atom sites substantially helps to understand the structure-property relationship in zeolites. The visibility of the oxygen atom was already attained in the raw OBF image before unit-cell averaging. The SNR of the S/TEM images of zeolites is usually enhanced by averaging the raw data using a priori knowledge about the sample, such as the space groups of the material (*27*, *28*). Although this is effective for homogeneous bulk structure analyses, it cannot be applied to heterogeneous or nonperiodic local structure analyses. Therefore, the presented direct atom imaging capability will be helpful for zeolitic heterogeneous/nonperiodic structure analyses, such as aluminum distribution in the framework T sites, extra-framework counter cations, and other defects. Later in this study, we have demonstrated the OBF STEM imaging of a defect structure in the FAU-type zeolite.

**Fig. 3.**
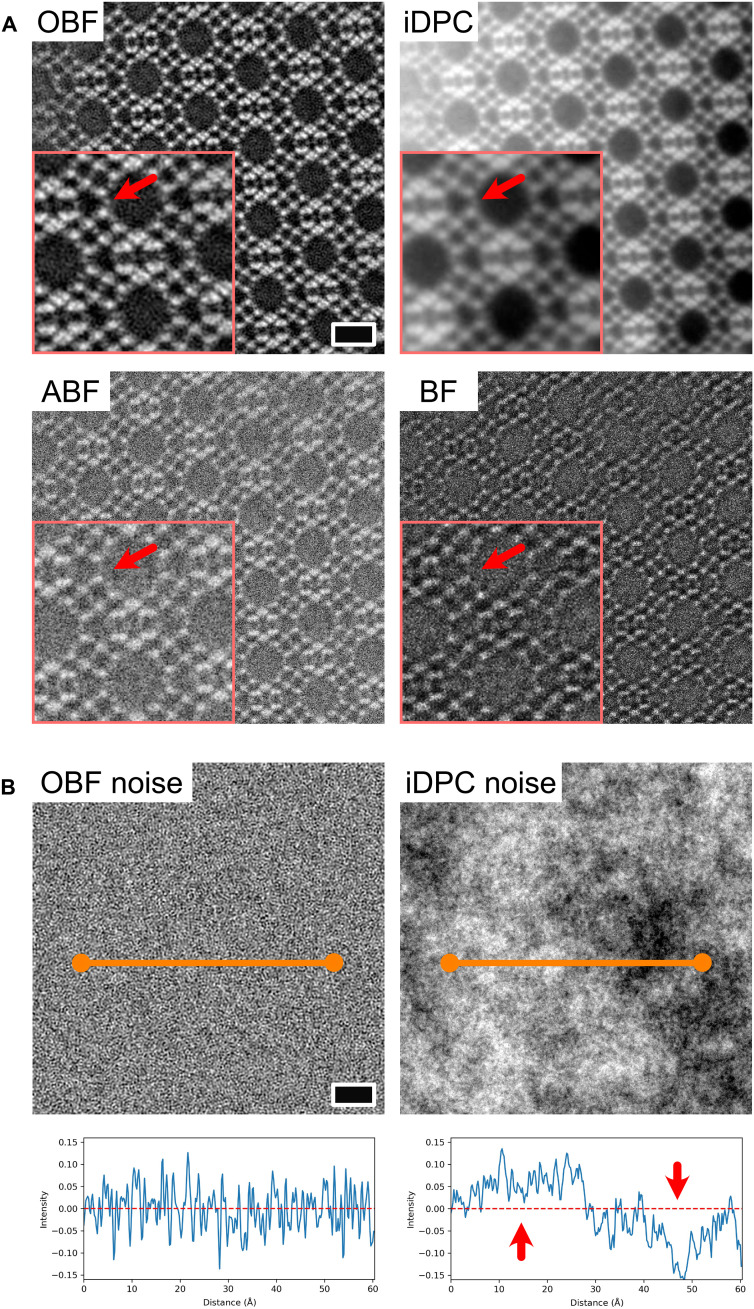
Comparison between atomic resolution images of OBF STEM and other STEM techniques. (**A**) STEM images obtained via OBF, iDPC, ABF, and conventional BF imaging techniques. Scale bar, 1 nm. All the images were recorded under the same electron dose and optical conditions (except for the defocus) as described in Materials and Methods. The insets are the cropped and enlarged versions of the original images, and the orange arrows indicate the oxygen sites in the FAU zeolitic structure. (**B**) Comparison of the noise components between the OBF and iDPC simulated images (see Materials and Methods for details). Scale bar, 1 nm. The intensity profiles of noise are also shown (obtained along the orange lines). The assumed dose is the same as that in the experiments shown in (A), and the noise components in both the methods are obtained by the same noise-introduced segmented detector datasets. As indicated by the orange line segments, the iDPC noise image has longer-range fluctuations than that of the OBF.

We compared the OBF images with other STEM images obtained under the same dose conditions. Figure 3A shows the OBF, iDPC, conventional BF, and ABF images. The OBF image shows the FAU framework structure with the highest SNR, conforming with the SNR transfer function calculation shown in Fig. 1B. Although the iDPC image also reveals the basic FAU framework structure, individual atomic sites, such as oxygen columns, are not distinguishable, as shown in the inset. For the further analysis of these contrast characteristics, we simulated the noise components of OBF and iDPC images (see Materials and Methods for details). In the OBF reconstruction, the noise level is set to be flat as a function of spatial frequency, which is known as the noise-normalized condition. This is equivalent to the so-called white noise. The noise fluctuation of the OBF image contrast is thus uniformly random for the entire FOV. This noise-component image is displayed on the same spatial scale as that of the experimental images shown in Fig. 3A for comparison. However, for the iDPC image, the contrast fluctuation due to noise exists on a spatially larger scale than that of the OBF image. This is confirmed by a line profile of the noise component. In the integration process of the DPC signal to form the iDPC image, the low-spatial-frequency component of the image is much more amplified than the higher-spatial-frequency components (*29*). However, the iDPC signals essentially do not exhibit contrast transfer around the low-frequency domains against noise, as shown in Fig. 1B. Thus, under the low-dose condition, this amplification effect enhances the noise component in the lower frequency regions, resulting in the appearance of long-range contrast fluctuation, as shown in Fig. 3B. This is the reason why the iDPC image contrast appears smoother but has longer-range noise fluctuation than those of the other methods. We also examined the experimental image intensity distribution of each imaging technique, as shown in fig. S1, wherein the longer-range noise effect was more severe owing to the wide FOV. Although the OBF image exhibits an interpretable image contrast corresponding to the sample thickness and atomic sites, the iDPC image exhibits long-range intensity fluctuations in the experiment and the simulations, as shown in Fig. 3B. This contrast is much stronger than that of each atomic site in the zeolitic framework. This results in a poor visibility of the atomic sites and makes it difficult to interpret the atomic structures from the obtained image. Recently, the iDPC techniques are used to observe zeolites including absorbed guest molecules and framework deformation due to the absorption (*18*–*20*). Because these phenomena occur in very localized region, the imaging property of iDPC technique as discussed above may affect the interpretation of the images, while the OBF could offer more reliable and interpretable image contrast with higher dose efficiency. In other STEM images, such as ABF and BF, the basic structure of the FAU framework is roughly visible, but the detailed atomic structure analysis is challenging under the present low-dose condition.

### Direct observation of FAU twin boundary

**Fig. 4.**
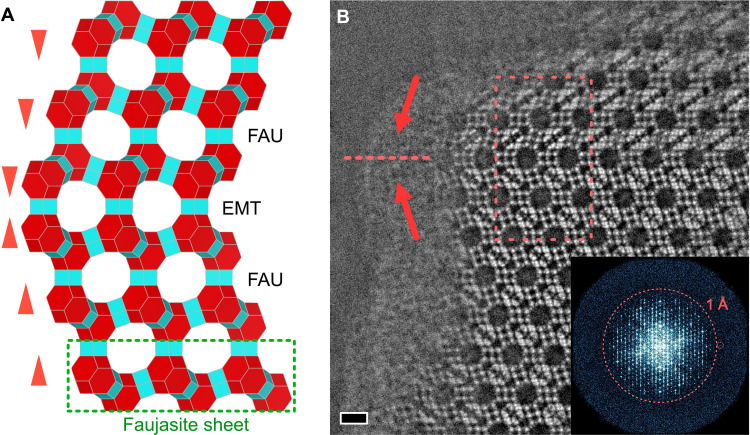
Atomic-resolution OBF STEM image of FAU twin boundary. (**A**) Framework model of the FAU twin boundary. The FAU cubic stacking sequence is inverted on the twin boundary, making the EMT framework structure with hexagonal stacking. The structure highlighted with a green dotted box is a faujasite sheet, a layer structure unit for the FAU and the EMT frameworks. The triangles represent the directions of the stacking sequence. (**B**) Atomic-resolution OBF STEM image of the FAU twin boundary. Scale bar, 1 nm. The inset is the FFT pattern of the OBF image, which exhibits a contrast transfer beyond 1 Å. The dashed rectangular indicates the repeat unit structure used for the averaging process shown in Fig. 5A.

We applied the OBF technique to characterize the atomic structure of a twin boundary in the FAU zeolite. In FAU-type zeolites, the framework is constructed by cubic stacking of a layered structure unit called a “faujasite sheet” (*30*). When the faujasite sheets are stacked in a hexagonal sequence, the resultant framework exhibits an EMT-type structure with lattice constant of *a* = *b* = 17.2150 Å, *c* = 28.0820 Å, α = β = 90°, and γ = 120° (space group *P*6_3_*/mmc*), known as a polymorph of an FAU-type zeolite. There are twin boundaries between two opposite sequences of the cubic stacking in the FAU framework that likely result in an EMT-type structure at the boundary, as schematically shown in Fig. 4A. However, the detailed atomic structure could not be directly determined owing to the limited spatial resolution under the low-dose condition in the previous TEM study (*31*).

**Fig. 5.**
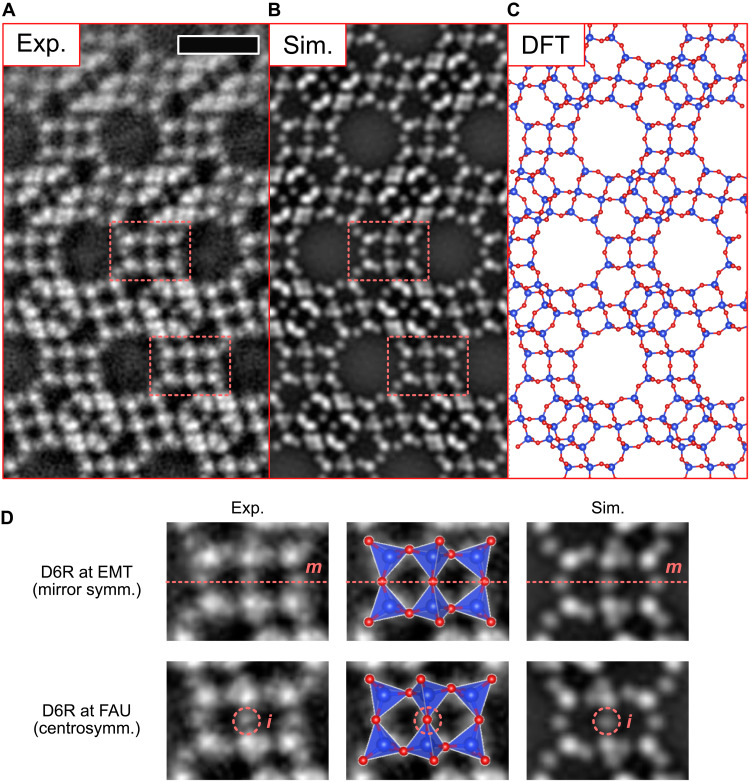
Comparison between experimental OBF image and simulated image based on DFT-relaxed structure of the FAU twin boundary. (**A**) Unit-cell–averaged experimental OBF image obtained from the raw experimental image shown in Fig. 4B. The averaging operation is performed along the direction parallel to the interface, and no structural information is assumed about the symmetry other than the translational symmetry along the boundary. Scale bar, 1 nm. (**B**) Simulated OBF image based on the DFT-relaxed structure shown in (C). The image is calculated under the same condition as that of the experiment. (**C**) Atomic structure model of FAU twin boundary relaxed by the DFT calculation. The blue and red balls indicate the T and oxygen sites, respectively. These images/structures show good agreement in both T and oxygen sites. (**D**) Comparison of D6R structures on the twin boundary and bulk region. Experimental and simulated images are shown for each structure, and atomic structure models represented as a TO_4_ tetrahedral configuration are also overlaid.

Figure 4B shows the OBF image of the FAU twin boundary. This image indicates that the FAU cubic stacking sequence is inverted at the twin boundary. The power spectrum of the image indicates an information transfer beyond 1 Å. For further analysis, we averaged the structural units of the FAU twin boundary, as shown in Fig. 5A. The T and oxygen atomic sites are evidently visible in the twin boundary core, and two FAU-type domains are connected coherently at the atomic scale. Looking more in detail at the observed D6R structures including the oxygen sites, it is evident that the atomic structure of D6Rs at the twin interface is different from the those in the FAU bulk structure. The D6Rs in FAU have centrosymmetry, which is observed in Fig. 2E as a projected configuration of oxygen atoms. Contrastingly, the observed D6Rs at the twin interface lack the centrosymmetry and instead exhibit mirror symmetry as shown in Fig. 5D. This observed symmetry at the twin interface is consistent with the D6Rs along the hexagonal stacking in the EMT-type structure. Because the T sites are almost identical for the two types of D6R, the structural change of D6R cannot be distinguished without oxygen sites. Therefore, low-dose light-element imaging such as OBF STEM is of much importance on structure analysis of zeolites including local change of symmetry as found here.

Density functional theory (DFT) calculations were performed to evaluate the stability of the twin boundary structure. The initial twin atomic structure was created by stacking the faujasite sheets, considering the local symmetries based on the OBF image, and then relaxed via DFT calculations. Figure 5C shows the relaxed atomic structure model, and Fig. 5B shows its corresponding simulated OBF image. The experimental image conforms well with its simulated counterpart. Furthermore, the interface energy was calculated to be 7.4 mJ/m^2^, which is comparable with those of the twin boundaries in face-centered cubic (FCC) metals on the {111} plane (*32*), but approximately three orders of magnitude lower than those (typically) in oxide ceramic materials, such as grain boundaries and twin boundaries (*33*, *34*). The origin of this difference can be explained as follows: In the {111} twin boundary of cubic zirconia, for example, the origin of the higher interface energy is attributed to the different coordination numbers of anions around the cation sites on the interface, whereas the cation sites produce a coherent interface structure similar to those of FCC metals (*35*). In the case of zeolites, the framework is constructed by the corner sharing of rigid TO_4_ tetrahedra, which have a nearly perfect tetrahedral shape and are connected via oxygen atoms as soft hinges, offering a rigid but stress-free atomic structure (*36*). Thus, zeolites can relax their framework structure by simply changing the bond angle between two rigid TO_4_ tetrahedrons (T-O-T angle). In silicate materials, the atomic structure is energetically stable over a wide range of T-O-T angles (*37*). The observed structure of the FAU twin boundary was constructed in a similar manner, keeping the coordination numbers of the cations and anions unchanged across the boundary. This structural flexibility should result in extremely low excess energy at the twin boundary. Structural information about minute strains around some defects is essential for applications such as molecular sieves and gas separators, where these guests and their dynamics are recently becoming possible to observe at the atomic level via in situ STEM techniques (*20*). It may affect the diffusion process of ions and molecules adsorbed in the zeolitic nanocavity.

### Application for a further beam-sensitive zeolite

In addition to the OBF STEM imaging of bulk and twin interface structures in FAU-type zeolite, we also applied this technique to a different type of zeolite sample (i.e., another Si/Al ratio and framework structure). In zeolitic frameworks, the Si/Al ratio and framework topology are crucial to the materials properties because the aluminum sites have negative charge that can capture cations and the shape of nano-sized pores influences the kind of guest species such as ions and molecules. Here, we applied the OBF technique to a Na-LTA type zeolite, which is one of the zeolites with a theoretically maximum aluminum content (Si/Al = 1) and the lowest framework density, being known as an extremely beam-sensitive zeolite (*27*). The Na-LTA zeolite has been investigated via HRTEM and ABF STEM combined with additional filtering or averaging processes, which need prior knowledge about crystallographic structure of the sample (*27*, *38*). Thus, we applied the low-dose OBF STEM technique for direct atomic observation of the Na-LTA zeolite sample.

**Fig. 6.**
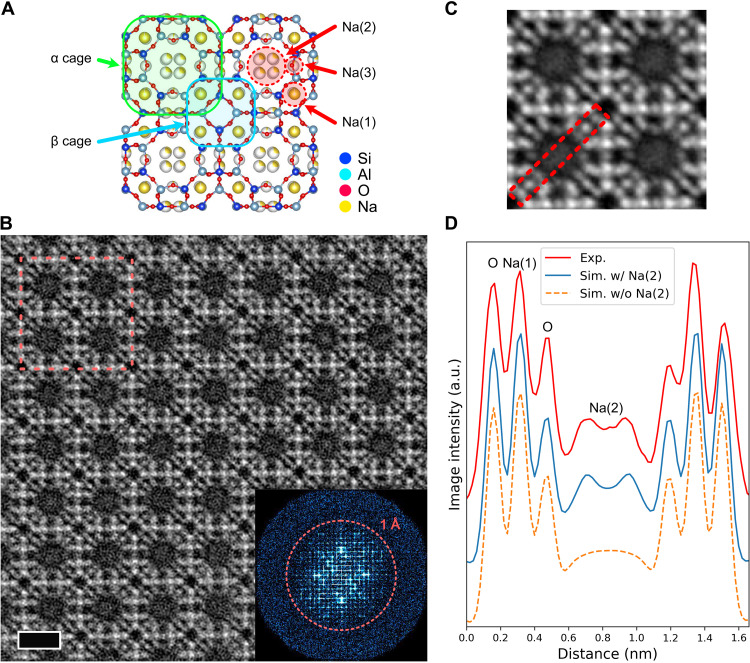
OBF STEM imaging of Na-LTA zeolite. (**A**) Atomic structure model of Na-LTA zeolite with Si/Al = 1 along the [001] direction. The LTA framework consists of two kinds of cages (α cage and β cage), and Na atoms are captured inside the framework. (**B**) OBF STEM image of Na-LTA zeolite sample. The inset is FFT pattern of the image where the orange dotted circle shows an information transfer of 1 Å. Scale bar, 1 nm. The dashed rectangular indicates the repeat unit structure used for the averaging process shown in (C). (**C**) Unit-averaged OBF image shown in (B). (**D**) Intensity profile of experimental and simulated OBF images for Na-LTA zeolite sample. The area for taking profiles is highlighted by dashed rectangular shown in (C). The simulated images are calculated for the atomic structure model with/without the Na(2) sites as shown in fig. S4. The intensities are shown in arbitrary units (a.u.).

Figure 6A shows the atomic structure model of Na-LTA zeolite along the [001] direction whose lattice constant is *a* = *b* = *c* = 24.555 Å and α = β = γ = 90° with a space group *Fm-*3*c* (*39*). The LTA-type framework structure consists of two types of cages (α cage and β cage), and Na^+^ ions are captured inside the frameworks occupying three different sites. We observed this sample with a probe current of 0.18 pA, which is much lower than that in the case of FAU-type sample shown in Fig. 2. Figure 6B shows the obtained raw OBF STEM image of the Na-LTA sample. Despite the very low-dose condition, the LTA framework structure is evidently visualized. Furthermore, information transfer around 1 Å is also confirmed via fast Fourier transform (FFT) pattern of the OBF image. We also investigated the unit-cell–averaged OBF image of Na-LTA zeolite, where no prior knowledge is assumed as discussed in Fig. 2. Figure 6C shows the unit-averaged OBF image, visualizing not only atomic sites inside the framework (Si, Al, and O sites) but also Na(1) sites captured in the β cage. Furthermore, the OBF image exhibits slight contrast inside the α cage, which should correspond to captured Na atoms. The intensity line profile obtained from the dashed rectangle is shown in Fig. 6D. Two distinct peaks are present inside the α cage. This result is consistent with the atomic structure based on a single-crystal x-ray diffraction (XRD) analysis shown in Fig. 6A (*39*), where the Na site in the α cage split into four sites, denoted as Na(2), and their site occupancy is 0.242 (~1/4). To investigate the consistency of the OBF image with the structure model based on XRD, we performed multislice simulations for the atomic structure models with/without Na(2) sites as shown in fig. S4. Figure 6D shows the intensity line profiles of the experimental image and simulated images with/without Na(2) sites. The experimental intensity profile agrees very well with simulated one with Na(2) sites quantitatively, which means that the OBF observation directly confirmed the Na-site splitting in the α cage, offering the capability of visualizing extra cation sites with low occupancy in zeolitic framework.

## DISCUSSION

We developed a highly dose-efficient STEM imaging technique, OBF STEM, for application in low-dose atomic-resolution imaging. We demonstrated that OBF STEM can directly reveal the atomic structures of all elements in an FAU-type zeolite, which is a beam-sensitive material, with a subangstrom spatial resolution. OBF STEM can also be used to observe the lattice defects in zeolitic framework structures. We succeeded in directly determining the atomic structure on an FAU twin boundary, and the corresponding result was consistent with the DFT calculations. Furthermore, we also observed Na-LTA type zeolite, which is one of the most beam-sensitive zeolite samples. We succeeded in visualizing not only all atomic sites in the framework but also the captured cations using OBF STEM in quantitative agreement with image simulations. The proposed technique can thus be used to characterize the local atomic structure in zeolites and other beam-sensitive materials, facilitating the study of structure-property relationships in these materials.

## MATERIALS AND METHODS

### Atomic-resolution OBF STEM observation of an FAU-type zeolite

Atomic-resolution OBF STEM images were acquired using an aberration-corrected STEM (JEOL JEM ARM-300F) equipped with a second-generation segmented annular all-field (SAAF) detector (16-segmented type) (*40*). We developed an in-house program for the real-time OBF display function and implemented it in the SAAF system, as shown in fig. S3. Movie S1 shows the real-time observation of a SrTiO_3_ [001] sample with an accelerating voltage of 300 kV and a probe-forming aperture of 30 mrad. All the atomic columns, including the oxygen atoms, were visualized under a low-dose condition (probe current: 0.5 pA, i.e., two orders of magnitude less than the usual condition). This result demonstrated the capability of OBF STEM for low-dose and live atomic-resolution imaging. We used a real-time OBF display system to acquire all the experimental OBF images shown in the present study.

For the TEM sample preparation of an FAU- and Na-LTA–type zeolites, commercially available powder samples of FAU zeolite (Tosoh Corp., Si/Al = 50) and Na-LTA zeolite (Tosoh Corp., Si/Al = 1) were gently crushed in an agate mortar with ethanol and dispersed onto a TEM microgrid. Before STEM observation, the sample was dehydrated overnight in the high-vacuum environment of the TEM column to suppress the irradiation damage (*8*). The accelerating voltage was set to 300 kV, which effectively reduces the irradiation damage in the zeolites (*6*, *41*), and probe-forming aperture was set to 15 mrad. The probe current was 0.5 pA for FAU zeolite and 0.18 pA for Na-LTA zeolite, respectively. Images of the FAU bulk structure were sequentially acquired at a dwell time of 16 μs with 1024 × 1024 pixels in the same region to suppress irradiation damage and scan distortion. Under this condition, the total dose was 1.2 ×10^3^ e^−^/Å^2^ per frame. For the FAU twin boundary observation, the dwell time was reduced to 10 μs to further suppress the image distortion, and the total dose was 7.5 ×10^2^ e^−^/Å^2^ per frame. For the Na-LTA zeolite observation, the dwell time was 5 μs, resulting in the total dose of 1.4 × 10^2^ e^−^/Å^2^ per frame. After the sequential image acquisition, the first five images for the FAU zeolite and the first eight images for the Na-LTA zeolite were aligned and averaged for each dataset. Furthermore, we obtained unit-cell–averaged images for a detailed structural analysis, as shown in Figs. 2D, 5A, and 6C. It can be noted that a priori knowledge about the structure group symmetry of the atomic structure was not assumed for the image averaging except for the translational symmetry for FAU bulk, FAU twin boundary, and Na-LTA analyses.

To obtain the OBF images, the camera length was set such that the edge of the STEM direct beam disk coincided with the outermost edge of the SAAF detector. Under these conditions, the OBF image was obtained using Eq. 1

$$I_{\mathrm{OBF}}(R_{p})=F^{-1}\left[\sum_{j=1}^{16}I_{j}(Q_{p})W_{j}(Q_{p})\right]=\sum_{j=1}^{16}I_{j}(R_{p})⊗w_{j}(R_{p})$$ (1)

where *I*_OBF_(***R***_p_), *I_j_*(***Q***_p_), *W_j_*(***Q***_p_), and *w_j_*(***R***_p_) are the OBF image intensity, Fourier-transformed image acquired by the *j*-th segment *I_j_*(***R***_p_), frequency filter calculated for the *j*-th segment, and point spread function obtained via the inverse Fourier transform of the frequency filter *W_j_*(***Q***_p_), respectively. The filtering process was performed by multiplying the frequency filter in the reciprocal space ***Q***_p_ or convolution with the point spread function in the real space ***R***_p_. The postprocessed OBF image could be obtained via either procedure, and the real-time OBF imaging synchronized with the STEM scan was acquired using the approximated convolution process in real space (*13*). For the focal condition to obtain the STEM images, it was reported that the OBF and DPC image contrast can be theoretically maximized upon focusing the electron probe on the mid-plane of the specimen (*13*, *42*). In contrast, the ABF and BF images exhibited the highest contrast upon focusing the probe on the entrance surface (*16*). Thus, we acquired the images under the optimal focal conditions for each technique. To obtain the experimental/simulated iDPC, BF, and ABF images, the segmented/annular detector images were synthesized using the SAAF detector datasets to reproduce the detector geometry dedicated to each method.

### Image simulations

For the STEM image simulation, we used the MuSTEM package (*43*) based on the multislice model (*44*). The 16-segmented detector images were calculated and processed using the OBF reconstruction algorithm to obtain the simulated OBF image. The effective source size was considered by convolution with a two-dimensional Gaussian with a full-width half maximum of 0.6 Å. The sample thickness was assumed to be 10 nm, and the defocus Δ*f* was set to middle-focus condition, wherein the focal plane is located at the mid-plane inside the sample (Δ*f* = −5 nm).

The STEM image simulation was also used for noise property analysis, as shown in Fig. 3B. First, noise was added to the simulated images of each detector segment based on the Poisson statistics. The noisy and noise-free images of each imaging method were then reconstructed. The assumed dose was equal to that of the experimental condition, as shown in Fig. 3A. The noise component images were then obtained by subtracting the noise-free images from their noisy counterparts, as shown in fig. S2. The noise-component images were normalized using the contrast range of their corresponding noise-free image. The noise characteristics of different techniques were then compared, as shown in Fig. 3B.

### DFT calculations

To relax the FAU twin-boundary structure and calculate the interface energy, we performed DFT calculations using the Vienna ab initio simulation package code (*45*) with the rev-vdW-DF2 method (*46*), which is suitable for calculating zeolitic atomic structures and energies (*47*). For the relaxation, we first relaxed the FAU bulk structure, whose data are available in the International Zeolite Association database (*48*). The initial FAU twin boundary structure was then created by connecting the two FAU framework models with opposite stacking sequences. Last, we obtained the relaxed FAU twin structure and calculated the interface energy Δ*E*_interface_ as follows

$$\text{Δ}E_{\mathrm{interface}}=\frac{E_{\mathrm{twin}}-E_{\mathrm{bulk}}}{2A}$$ (2)

where *A* is the cross-sectional area of the interface and *E*_bulk_ and *E*_twin_ are the total energies of the FAU bulk and twin boundary structures, respectively.

## Supplementary Materials

### This PDF file includes:

Supplementary Text Figs. S1 to S5 Table S1 Legend for movie S1 References
