# Supplementary material for: Direct imaging of local atomic structures in zeolite using optimum bright-field scanning transmission electron microscopy
Source: Sci Adv. 2023 Aug 2;9(31):eadf6865. doi: 10.1126/sciadv.adf6865 (PMC10396294; doi:10.1126/sciadv.adf6865)
Supplement: Supplementary file 1 — Supplementary Text Figs. S1 to S5 Table S1 Legend for movie S1 References [file sciadv.adf6865_sm.pdf]

Science Advances
AAAS

Supplementary Materials for

Direct imaging of local atomic structures in zeolite using optimum bright-field scanning transmission electron microscopy

Kousuke Ooe *et al.*

Corresponding author: Takehito Seki, seki@sigma.t.u-tokyo.ac.jp; Naoya Shibata, shibata@sigma.t.u-tokyo.ac.jp

Sci. Adv. **9**, eadf6865 (2023)
DOI: 10.1126/sciadv.adf6865

The PDF file includes:

Supplementary Text
Figs. S1 to S5
Table S1
Legend for movie S1
References

Other Supplementary Material for this manuscript includes the following:

Movie S1

Supplementary Text

<u>Basic concept of SNR transfer functions</u>

In Figs. 1B and S5, we evaluated dose efficiency of different STEM techniques by calculating SNR transfer functions, which is defined as CTF divided by noise level. In this note, we describe the basic concept of the SNR transfer function.

In STEM, a normal CTF represents only the transfer characteristic from the electrostatic potential of a specimen to image contrast as:

$$\mathcal{F}\left[I_{\mathrm{STEM}}(\boldsymbol{R}_{\mathrm{p}})\right]\left(\boldsymbol{Q}_{\mathrm{p}}\right) = d_0\delta\left(\boldsymbol{Q}_{\mathrm{p}}\right) + \beta\left(\boldsymbol{Q}_{\mathrm{p}}\right)\sigma V\left(\boldsymbol{Q}_{\mathrm{p}}\right), \tag{S1}$$

where $\mathcal{F}$, $I_{\mathrm{STEM}}(\boldsymbol{R}_{\mathrm{p}})$, d_0, $\delta\left(\boldsymbol{Q}_{\mathrm{p}}\right)$, $\beta\left(\boldsymbol{Q}_{\mathrm{p}}\right)$ and $\sigma V\left(\boldsymbol{Q}_{\mathrm{p}}\right)$ represent the Fourier transform operator, the reconstructed image, the background intensity in real space, the delta function, the normal CTF and Fourier transform of the electrostatic potential of the specimen multiplied by the interaction constant, respectively ($\boldsymbol{R}_{\mathrm{p}}$ and $\boldsymbol{Q}_{\mathrm{p}}$ are two-dimensional real-space coordinate and its conjugate spatial frequency coordinate). Since the normal CTF does not consider the noise propagation in the reconstruction process, it is impossible to compare different STEM techniques in terms of dose efficiency. To compare different STEM techniques, we first developed the noise-evaluation theory for arbitrary linear reconstruction processes (15). To calculate the signal-to-noise ratio of the reconstructed image based on the theory, both sides of Eq. (S1) should be divided by the noise level as a function of the spatial frequency $N\left(\boldsymbol{Q}_{\mathrm{p}}\right)$:

$$\mathcal{F}\left[I_{\mathrm{STEM}}(\boldsymbol{R}_{\mathrm{p}})\right]\left(\boldsymbol{Q}_{\mathrm{p}}\right)/N(\boldsymbol{Q}_{\mathrm{p}}) = \left[\beta(\boldsymbol{Q}_{\mathrm{p}})/N(\boldsymbol{Q}_{\mathrm{p}})\right]\sigma V(\boldsymbol{Q}_{\mathrm{p}}). \tag{S2}$$

For simplicity, we described the Fourier components other than the DC component ($\boldsymbol{Q}_{\mathrm{p}} = 0$). Here, we define the SNR transfer function as $\beta(\boldsymbol{Q}_{\mathrm{p}})/N(\boldsymbol{Q}_{\mathrm{p}})$. Then, the product of SNR transfer function and the Fourier transform of the electrostatic potential (the right side of Eq. (S2)) equals to the SNR of the measured Fourier components (the left side of Eq. (S2)). In practice, since the noise level depends on the electron dose, the SNR transfer function is defined as $\beta(\boldsymbol{Q}_{\mathrm{p}})/n(\boldsymbol{Q}_{\mathrm{p}})$, where $n\left(\boldsymbol{Q}_{\mathrm{p}}\right)$ is the dose-invariant noise factor. Details are already discussed in the previous paper (15), but usually $N\left(\boldsymbol{Q}_{\mathrm{p}}\right) = n\left(\boldsymbol{Q}_{\mathrm{p}}\right)/\sqrt{n_e}$, where n_e denotes electron dose. Thus, the SNR transfer functions become proportional to the SNR of measured Fourier components, which is powerful parameter for fairly comparing the dose-efficiency of different STEM techniques when the same specimen is observed at the same dose condition. More detailed discussions such as calculation of $n\left(\boldsymbol{Q}_{\mathrm{p}}\right)$ for the linear reconstruction processes can be found in the previous literatures (13, 15), where the SNR transfer function is referred to as “noise-normalized CTF”.

Dose efficiency evaluation based on the SNR transfer functions

To compare the contrast transfer efficiency of different STEM techniques against the noise-level, we calculated the SNR transfer functions as discussed above. In this note, we show how to evaluate the theoretical dose efficiency of each technique by the SNR transfer functions as shown in Table S1.

By taking the inverse Fourier transform of Eq. (S1), the STEM image intensity $I_{\mathrm{STEM}}(\boldsymbol{R}_{\mathrm{p}})$ is given as follows

$$I_{\mathrm{STEM}}(\boldsymbol{R}_{\mathrm{p}}) = d_0 + \mathcal{F}^{-1}[\sigma V(\boldsymbol{Q}_{\mathrm{p}})\beta(\boldsymbol{Q}_{\mathrm{p}})], \tag{S3}$$

where $\mathcal{F}^{-1}$ indicates the inverse Fourier transformation operator. Assuming that the specimen projected potential in real space $v(\boldsymbol{R}_{\mathrm{p}})$ is a delta function (i.e., $v(\boldsymbol{R}_{\mathrm{p}}) \cong v_0\delta(\boldsymbol{R}_{\mathrm{p}})$ where v_0 is a constant), the STEM image intensity above the point scatterer ($\boldsymbol{R}_{\mathrm{p}} = \mathbf{0}$) $I_{\mathrm{STEM}}(\mathbf{0})$ is approximated as follows

$$I_{\mathrm{STEM}}(\mathbf{0}) = d_0 + \sigma v_0 \int \beta(\boldsymbol{Q}_{\mathrm{p}})\, \mathrm{d}\boldsymbol{Q}_{\mathrm{p}}. \tag{S4}$$

This equation indicates that we can evaluate the contrast amplitude approximately by the second term, integration of CTF over the frequency domains, because the first term d_0 is the background. Thus, we compare the obtainable contrast against the noise-level among different STEM techniques by integrating the SNR transfer functions (*13*, *50*). In the Poisson statistics, the signal-to-noise ratio (SNR) is proportional to the $\sqrt{\lambda}$, where λ is the electron dose. Here, the integration value of SNR transfer functions can be regarded as a relative SNR between different techniques, and thus we can compare the dose-efficiency by the squared values of the integration of SNR transfer functions.

Firstly, we calculated SNR transfer functions for the methods using not only segmented or conventional detectors (shown in Fig. 1B) but also for the methods using a pixelated detector such as single side band (SSB) ptychography (*51*) and iCoM (integrated center-of-mass) (*52*, *53*) as shown in Fig. S5. The iCoM method is a kind of iDPC imaging with a pixelated detector. Since we previously showed that OBF imaging can be extended to the pixelated detector (*13*), we also calculated the SNR transfer function of OBF using the pixelated detector. As for the segmented detector, the detector shape is the same as the previous literatures for OBF (*40*) and iDPC (*17*), respectively. Then, the dose efficiency ratio was calculated by the integration of the SNR transfer functions for each technique as shown in Table S1. According to the calculated values shown in Table S1, OBF has approximately two-orders of magnitude higher dose efficiency than ABF theoretically. Furthermore, because OBF reconstructs the phase-contrast image in a theoretically optimized manner to obtain the highest SNR for each type of detector, the calculated dose efficiency of OBF is higher than the other techniques that use segmented or pixelated detectors, respectively. As for the pixelated detector, it is noted that OBF and SSB ptychography show close SNR transfer functions and dose-efficiency while the efficiency of both techniques is higher than the iCoM. From the theoretical aspect, pixelated detector OBF can be considered as the extension of SSB ptychography (13). This is because OBF extracts phase-contrast components in an efficient way under the thick weak phase object approximation (tWPOA) model, where the probe propagation inside the sample

thickness is taken into account, while SSB ptychography collects phase information under the projected approximation. This effect becomes substantial when the depth focus of the probe is smaller than the sample thickness because the phase of incident electron changes rapidly according to the defocus difference within the sample thickness. However, under the observation condition of this study, the depth focus (*54*) is estimated to be 8.7 nm (accelerating voltage of 300 kV and probe forming aperture of 15 mrad), which is comparable to the typical TEM samples for atomic-resolution experiments. Therefore, SNR transfer functions and dose-efficiency of pixelated detector OBF and SSB ptychography are somewhat similar, and obtainable images of both techniques should offer alike contrast especially in the case of performing the noise-normalization process in SSB ptychography (*55*). However, even in the relatively large depth focus condition, OBF with pixelated detector shows higher efficiency than SSB. Furthermore, this superiority becomes much substantial in the case of sharp depth focus (i.e., higher accelerating voltage and larger convergence angle). Additionally, the fast dwell time of STEM probe (approximately 1~10 μs) is recently available with the state-of-the-art pixelated detectors (*21*), and real-time SSB ptychographic reconstruction process of pixelated detector datasets are intensively investigated for realizing live imaging (*56*, *57*). Applying this scheme to the OBF, the pixelated detector OBF image can also be performed in real-time. In the present study, we used a segmented detector with live OBF imaging system and successfully observed beam-sensitive zeolite samples. Thus, real-time OBF imaging with the next-generation pixelated detector should also be definitely helpful for the further beam-sensitive material analysis. As for the segmented detector, while the dose-efficiency is slightly lower compared with the pixelated detector for each method, OBF has high dose efficiency than iDPC technique, which is comparable to iCoM using a pixelated detector. Since the iDPC technique is currently used for the STEM observation of beam-sensitive samples (*58*, *59*), the OBF observation should be able to reduce the irradiation dose more or obtain a higher spatial resolution on the same samples.

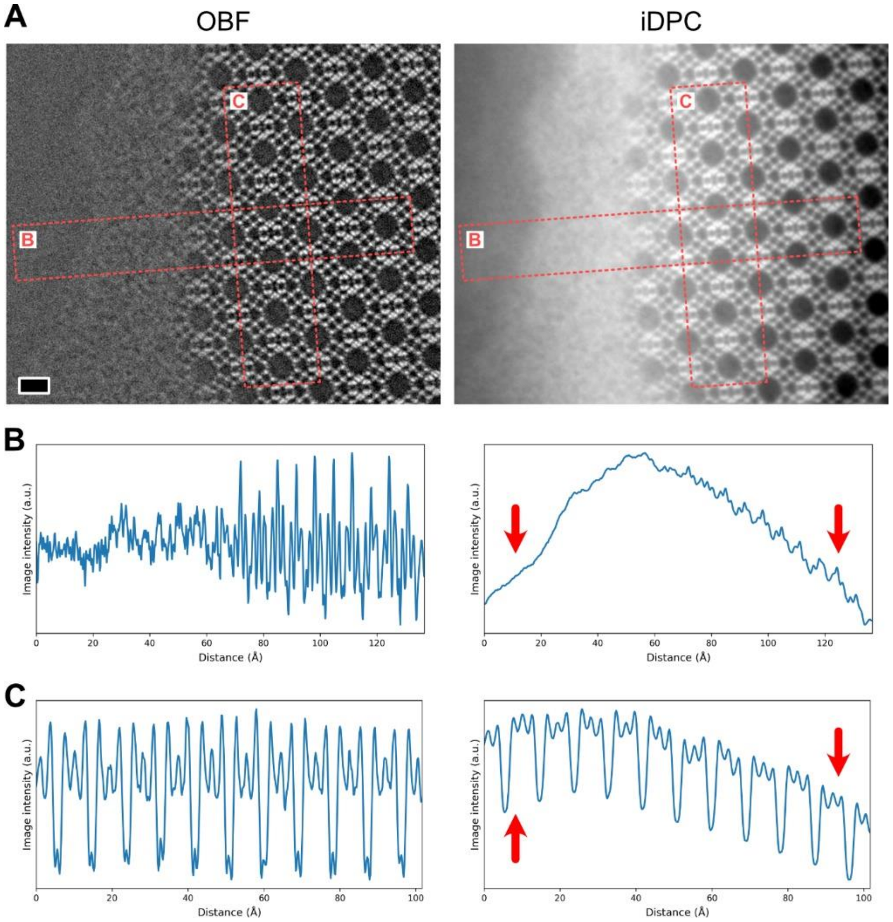

Fig. S1. Comparison between OBF and iDPC images obtained from the same experimental dataset. (A) OBF and iDPC images generated from the same dataset. The OBF image is the same as Fig. 2B (scale bar: 1 nm). The intensity profiles taken along (B) [001] direction and (C) [1-10] direction respectively from the orange rectangles shown in the OBF and iDPC images. Since the observed sample is wedge-shaped and has an amorphous layer near the edge, the projected atomic potentials should be increased from left hand side (vacuum area) to right hand side (thicker sample area) along the direction shown in (B). On the other hand, along the direction shown in (C), the thickness is almost uniform and corresponding image contrast should also be uniform. These are the case for the OBF image, but the iDPC image has strong intensity fluctuations (indicated by orange arrows) as discussed in Fig. 3B.

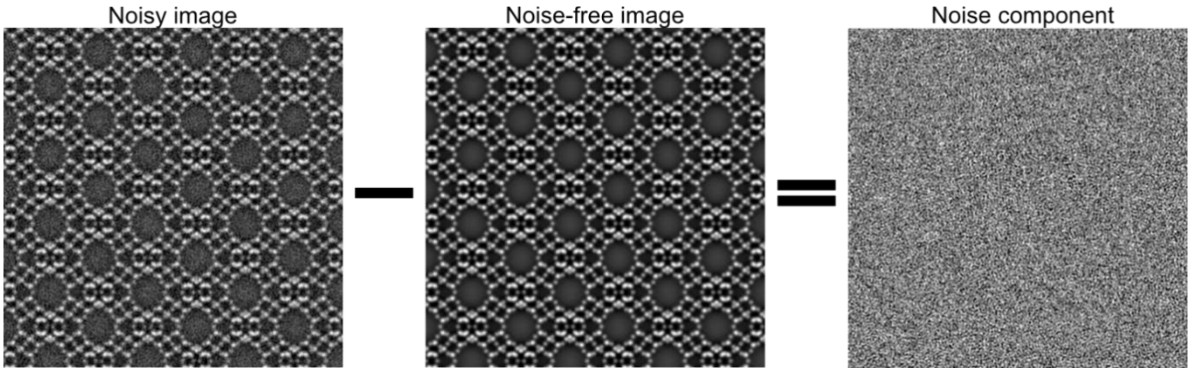

Fig. S2. Schematic of noise evaluation technique. Schematic illustration of noise evaluation method shown in Fig. 3B. By combining the noise-free image and noisy image, noise characteristics against the contrast range can be calculated.

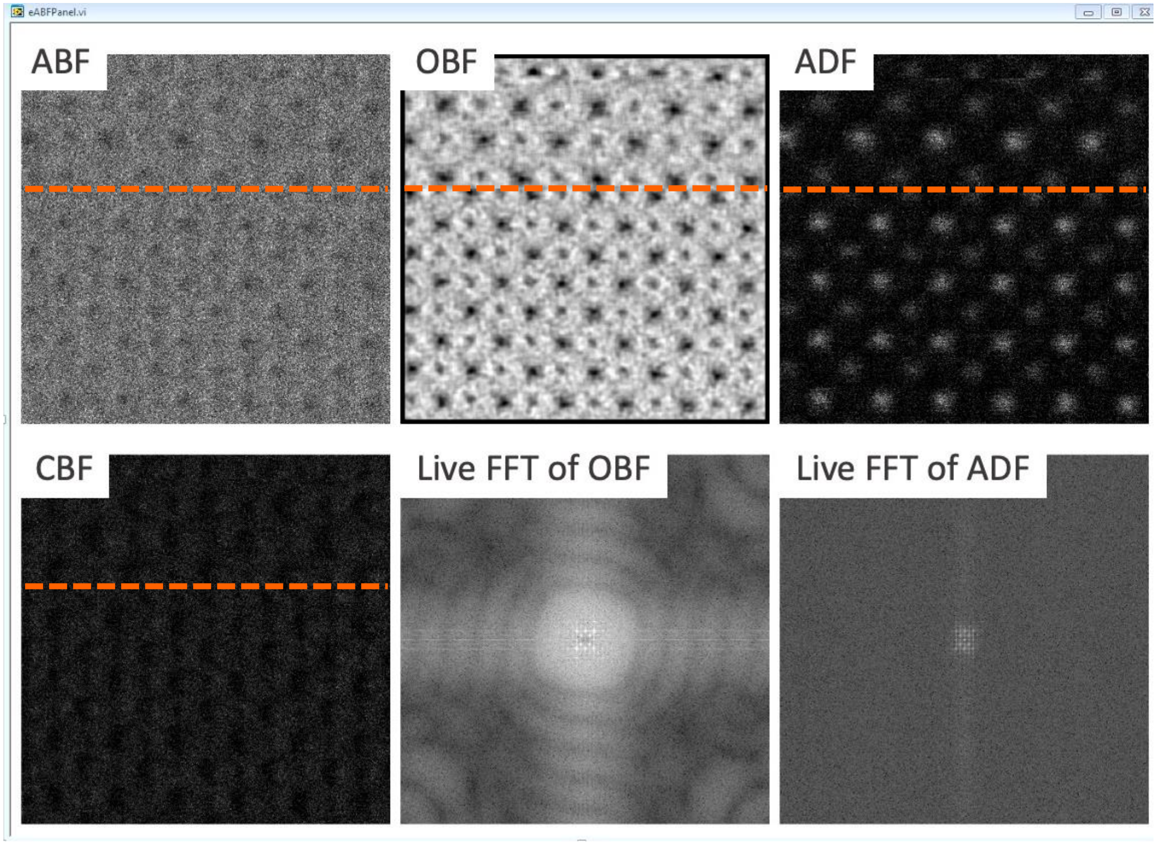

Fig. S3. Display of live OBF imaging system. Captured image of Movie S1 and its description. Movie S1 shows the real-time atomic-resolution OBF imaging of $SrTiO_3$ [001] under a low-dose condition. The dwell time is 10 µs, and the image is sampled with 512x512 pixels. In the upper row, the left panel, center panel, and the right panel show ABF, OBF, and annular dark-field (ADF) images, respectively. In the lower row, the left panel shows a center bright-field (CBF) image, and the center and right panel shows Fourier transformed OBF and ADF images, respectively. The images, including OBF, are synchronized with STEM probe scans and updated in real-time. The updated area in this capture is highlighted with a dotted line in each image. Movie S1 also shows the area scan mode, where the only selected area inside the image is scanned and the frame rate is increased for tuning aberrations such as defocus.

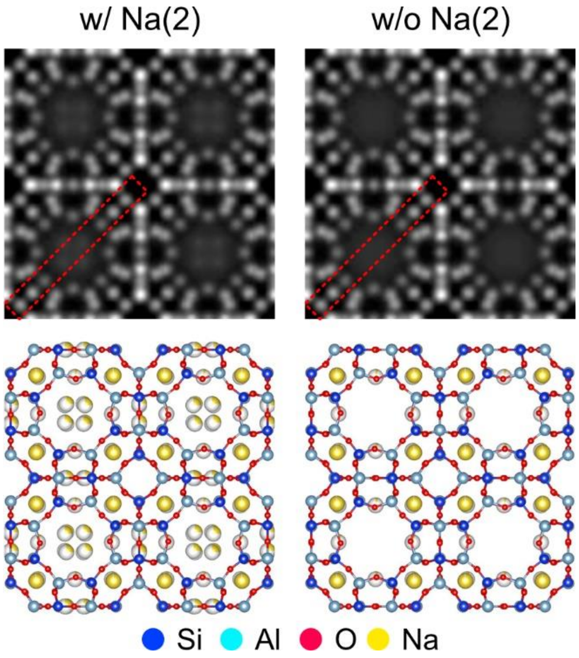

Fig. S4. Simulated OBF images for LTA zeolite with/without Na(2) sites. Simulated OBF images calculated for two atomic structures (with Na(2) sites and without Na(2) sites). The structure models are also shown, and dotted rectangular indicates the area for taking line profiles shown in Fig. 6D.

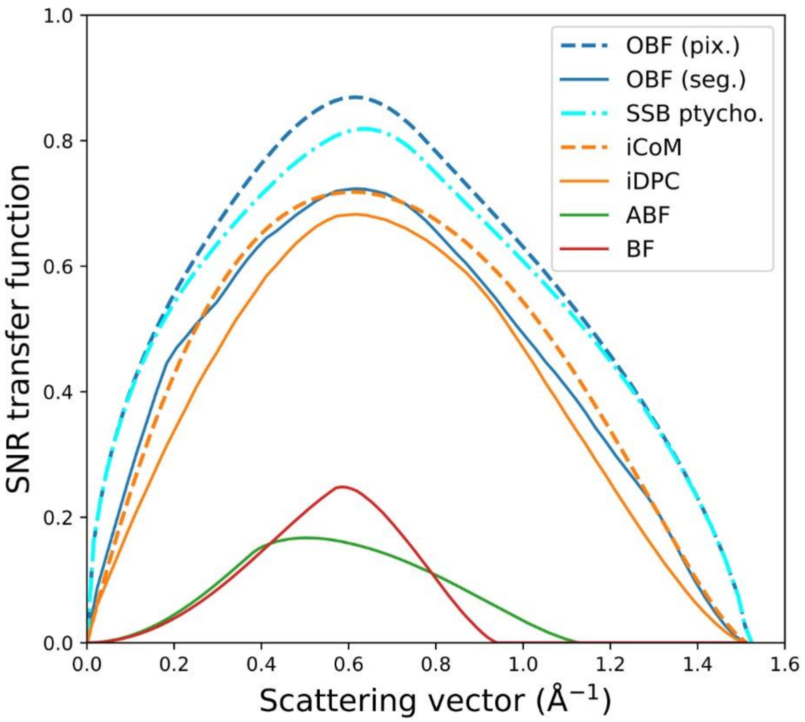

Fig. S5. SNR transfer functions including phase imaging techniques using a pixelated detector. SNR transfer functions for OBF using a pixelated and segmented detector, single side band (SSB) ptychography, iCoM, iDPC, ABF, and BF techniques. The transfer functions for OBF (seg.), iDPC, ABF, and BF are the same as the Fig. 1B. The plotted values are calculated as radially-averaged transfer functions.

Table S1. Comparison of dose efficiency of different STEM methods. Dose efficiency ratio among different STEM imaging techniques based on the SNR transfer function calculations. The values are normalized such that the dose-efficiency of ABF becomes one.

Detector type	Method	Dose-efficiency ratio
Pixelated	OBF	110
	SSB ptycho.	106
	iDPC (iCoM)	69.9
Segmented	OBF	66.5
	iDPC	53.8
Annular (conventional)	BF	1.02
	ABF	1

Movie S1.

Live atomic-resolution OBF imaging of $SrTiO_3$ [001] using the real-time OBF system.

REFERENCES AND NOTES

1. Y. Li, L. Li, J. Yu, Applications of zeolites in sustainable chemistry. *Chem* **3**, 928–949 (2017).

2. Z. Wang, J. Yu, R. Xu, Needs and trends in rational synthesis of zeolitic materials. *Chem. Soc. Rev.* **41**, 1729–1741 (2012).

3. Y. Li, J. Yu, New stories of zeolite structures: Their descriptions, determinations, predictions, and evaluations. *Chem. Rev.* **114**, 7268–7316 (2014).

4. S. Morishita, R. Ishikawa, Y. Kohno, H. Sawada, N. Shibata, Y. Ikuhara, Attainment of 40.5 pm spatial resolution using 300 kV scanning transmission electron microscope equipped with fifth-order aberration corrector. *Microscopy* **67**, 46–50 (2018).

5. S. X. Wang, L. M. Wang, R. C. Ewing, Electron and ion irradiation of zeolites. *J. Nucl. Mater.* **278**, 233–241 (2000).

6. O. Ugurlu, J. Haus, A. A. Gunawan, M. G. Thomas, S. Maheshwari, M. Tsapatsis, K. A. Mkhoyan, Radiolysis to knock-on damage transition in zeolites under electron beam irradiation. *Phys. Rev. B* **83**, 113408 (2011).

7. J. W. Menter, The electron microscopy of crystal lattices. *Adv. Phys.* **7**, 299–348 (1958).

8. L. A. Bursill, E. A. Lodge, J. M. Thomas, Zeolitic structures as revealed by high-resolution electron microscopy. *Nature* **286**, 111–113 (1980).

9. M. Haider, S. Uhlemann, E. Schwan, H. Rose, B. Kabius, K. Urban, Electron microscopy image enhanced. *Nature* **392**, 768–769 (1998).

10. C. Li, Q. Zhang, A. Mayoral, Ten years of aberration corrected electron microscopy for ordered nanoporous materials. *ChemCatChem* **12**, 1248–1269 (2020).

11. T. Seki, Y. Ikuhara, N. Shibata, Toward quantitative electromagnetic field imaging by differential-phase-contrast scanning transmission electron microscopy. *Microscopy* **70**, 148–160 (2021).

12. H. Yang, I. MacLaren, L. Jones, G. T. Martinez, M. Simson, M. Huth, H. Ryll, H. Soltau, R. Sagawa, Y. Kondo, C. Ophus, P. Ercius, L. Jin, A. Kovács, P. D. Nellist, Electron ptychographic phase imaging of light elements in crystalline materials using Wigner distribution deconvolution. *Ultramicroscopy* **180**, 173–179 (2017).

13. K. Ooe, T. Seki, Y. Ikuhara, N. Shibata, Ultra-high contrast STEM imaging for segmented/pixelated detectors by maximizing the signal-to-noise ratio. *Ultramicroscopy* **220**, 113133 (2021).

14. H. Rose, Nonstandard imaging methods in electron microscopy. *Ultramicroscopy* **2**, 251–267 (1977).

15. T. Seki, Y. Ikuhara, N. Shibata, Theoretical framework of statistical noise in scanning transmission electron microscopy. *Ultramicroscopy* **193**, 118–125 (2018).

16. S. D. Findlay, N. Shibata, H. Sawada, E. Okunishi, Y. Kondo, Y. Ikuhara, Dynamics of annular bright field imaging in scanning transmission electron microscopy. *Ultramicroscopy* **110**, 903–923 (2010).

17. E. Yücelen, I. Lazić, E. G. T. Bosch, Phase contrast scanning transmission electron microscopy imaging of light and heavy atoms at the limit of contrast and resolution. *Sci. Rep.* **8**, 2676 (2018).

18. B. Shen, H. Wang, H. Xiong, X. Chen, E. G. T. Bosch, I. Lazić, W. Qian, F. Wei, Atomic imaging of zeolite-confined single molecules by electron microscopy. *Nature* **607**, 703–707 (2022).

19. B. Shen, X. Chen, H. Wang, H. Xiong, E. G. T. Bosch, I. Lazić, D. Cai, W. Qian, S. Jin, X. Liu, Y. Han, F. Wei, A single-molecule van der Waals compass. *Nature* **592**, 541–544 (2021).

20. H. Xiong, Z. Liu, X. Chen, H. Wang, W. Qian, C. Zhang, A. Zheng, F. Wei, In situ imaging of the sorption-induced subcell topological flexibility of a rigid zeolite framework. *Science* **376**, 491–496 (2022).

21. D. Jannis, C. Hofer, C. Gao, X. Xie, A. Béché, T. J. Pennycook, J. Verbeeck, Event driven 4D STEM acquisition with a Timepix3 detector: Microsecond dwell time and faster scans for high precision and low dose applications. *Ultramicroscopy* **233**, 113423 (2022).

22. N. Shibata, T. Seki, G. Sánchez-Santolino, S. D. Findlay, Y. Kohno, T. Matsumoto, R. Ishikawa, Y. Ikuhara, Electric field imaging of single atoms. *Nat. Commun.* **8**, 15631 (2017).

23. Y. Sasaki, T. Suzuki, Y. Ikuhara, A. Saji, Direct observation of channel structures in zeolite Y and A with a slow-scan, charge-coupled-device camera. *J. Am. Ceram. Soc.* **78**, 1411–1413 (1995).

24. V. Alfredsson, T. Ohsuna, O. Terasaki, J.-O. Bovin, Investigation of the surface structure of the zeolites FAU and EMT by high-resolution transmission electron microscopy. *Angew. Chemie Int. Ed. English.* **32**, 1210–1213 (1993).

25. M. Shamzhy, M. Opanasenko, P. Concepción, A. Martínez, New trends in tailoring active sites in zeolite-based catalysts. *Chem. Soc. Rev.* **48**, 1095–1149 (2019).

26. M.-L. U. Cornelius, L. Price, S. A. Wells, L. F. Petrik, A. Sartbaeva, The steric influence of extra-framework cations on framework flexibility: An LTA case study. *Zeitschrift für Krist. Cryst. Mater.* **234**, 461–468 (2019).

27. A. Mayoral, Q. Zhang, Y. Zhou, P. Chen, Y. Ma, T. Monji, P. Losch, W. Schmidt, F. Schüth, H. Hirao, J. Yu, O. Terasaki, Direct atomic-level imaging of zeolites: Oxygen, sodium in Na-LTA and iron in Fe-MFI. *Angew. Chemie Int. Ed.* **59**, 19510–19517 (2020).

28. Y. Zhang, D. Smith, J. E. Readman, A. Mayoral, Direct imaging and location of Pb^{2+} and K^{+} in EMT framework-type zeolite. *J. Phys. Chem. C* **125**, 6461–6470 (2021).

29. H. G. Brown, N. Shibata, H. Sasaki, T. C. Petersen, D. M. Paganin, M. J. Morgan, S. D. Findlay, Measuring nanometre-scale electric fields in scanning transmission electron microscopy using segmented detectors. *Ultramicroscopy* **182**, 169–178 (2017).

30. J. M. Newsam, M. M. J. Treacy, D. E. W. Vaughan, K. G. Strohmaier, W. J. Mortier, The structure of zeolite ZSM-20: Mixed cubic and hexagonal stackings of faujasite sheets. *J. Chem. Soc. Chem. Commun.*, 493–495 (1989).

31. Y. Sasaki, T. Suzuki, Y. Takamura, A. Saji, H. Saka, Structure analysis of the mesopore in dealuminated zeolite Y by high resolution TEM observation with slow scan CCD camera. *J. Catal.* **178**, 94–100 (1998).

32. J. Rittner, D. Seidman, $\langle 110 \rangle$ symmetric tilt grain-boundary structures in Fcc metals with low stacking-fault energies. *Phys. Rev. B.* **54**, 6999–7015 (1996).

33. Y. Sato, T. Mizoguchi, F. Oba, Y. Ikuhara, T. Yamamoto, Arrangement of multiple structural units in a [0001] Σ49 tilt grain boundary in ZnO. *Phys. Rev. B.* **72**, 064109 (2005).

34. S. Fabris, S. Nufer, C. Elsässer, T. Gemming, Prismatic $\Sigma 3(10\bar{1}0)$ twin boundary in $\alpha{-}Al_2O_3$ investigated by density functional theory and transmission electron microscopy. *Phys. Rev. B.* **66**, 155415 (2002).

35. N. Shibata, F. Oba, T. Yamamoto, Y. Ikuhara, Structure, energy and solute segregation behaviour of [110] symmetric tilt grain boundaries in yttria-stabilized cubic zirconia. *Philos. Mag.* **84**, 2381–2415 (2004).

36. M. V. Chubynsky, M. F. Thorpe, Self-organization and rigidity in network glasses. *Curr. Opin. Solid State Mater. Sci.* **5**, 525–532 (2001).

37. C. J. Dawson, R. Sanchez-Smith, P. Rez, M. O’Keeffe, M. M. J. Treacy, Ab initio calculations of the energy dependence of Si-O-Si angles in silica and Ge-O-Ge angles in germania crystalline systems. *Chem. Mater.* **26**, 1523–1527 (2014).

38. K. Yoshida, K. Toyoura, K. Matsunaga, A. Nakahira, H. Kurata, Y. H. Ikuhara, Y. Sasaki, Structural analyses of sodium cations embedded within zeolitic nanocavities. *Microporous Mesoporous Mater.* **259**, 195–202 (2018).

39. J. J. Pluth, J. V Smith, Accurate redetermination of crystal structure of dehydrated zeolite A. Absence of near zero coordination of sodium. Refinement of silicon,aluminum-ordered superstructure. *J. Am. Chem. Soc.* **102**, 4704–4708 (1980).

40. N. Shibata, Y. Kohno, S. D. Findlay, H. Sawada, Y. Kondo, Y. Ikuhara, New area detector for atomic-resolution scanning transmission electron microscopy. *J. Electron Microsc. (Tokyo)* **59**, 473–479 (2010).

41. K. Yoshida, Y. Sasaki, Optimal accelerating voltage for HRTEM imaging of zeolite. *Microscopy* **62**, 369–375 (2013).

42. R. Close, Z. Chen, N. Shibata, S. D. Findlay, Towards quantitative, atomic-resolution reconstruction of the electrostatic potential via differential phase contrast using electrons. *Ultramicroscopy* **159**, 124–137 (2015).

43. L. J. Allen, A. J. D'Alfonso, S. D. Findlay, Modelling the inelastic scattering of fast electrons. *Ultramicroscopy* **151**, 11–22 (2015).

44. J. M. Cowley, A. F. Moodie, The scattering of electrons by atoms and crystals. I. A new theoretical approach. *Acta Crystallogr.* **10**, 609–619 (1957).

45. G. Kresse, J. Furthmüller, Efficient iterative schemes for ab initio total-energy calculations using a plane-wave basis set. *Phys. Rev. B.* **54**, 11169–11186 (1996).

46. I. Hamada, van der Waals density functional made accurate. *Phys. Rev. B* **89**, 121103 (2014).

47. M. Fischer, W. J. Kim, M. Badawi, S. Lebègue, Benchmarking the performance of approximate van der Waals methods for the structural and energetic properties of SiO_2 and $AlPO_4$ frameworks. *J. Chem. Phys.* **150**, 094102 (2019).

48. C. Baerlocher, L. B. McCusker, Database of zeolite structures (2017); www.iza-structure.org/databases/.

49. K. Momma, F. Izumi, *VESTA 3* for three-dimensional visualization of crystal, volumetric and morphology data. *J. Appl. Cryst.* **44**, 1272–1276 (2011).

50. K. Ooe, T. Seki, Y. Ikuhara, N. Shibata, High contrast STEM imaging for light elements by an annular segmented detector. *Ultramicroscopy* **202**, 148–155 (2019).

51. T. J. Pennycook, A. R. Lupini, H. Yang, M. F. Murfitt, L. Jones, P. D. Nellist, Efficient phase contrast imaging in STEM using a pixelated detector. Part 1: Experimental demonstration at atomic resolution. *Ultramicroscopy* **151**, 160–167 (2015).

52. K. Müller, F. F. Krause, A. Béché, M. Schowalter, V. Galioit, S. Löffler, J. Verbeeck, J. Zweck, P. Schattschneider, A. Rosenauer, Atomic electric fields revealed by a quantum mechanical approach to electron picodiffraction. *Nat. Commun.* **5**, 5653 (2014).

53. I. Lazić, E. G. T. Bosch, S. Lazar, Phase contrast STEM for thin samples: Integrated differential phase contrast. *Ultramicroscopy* **160**, 265–280 (2016).

54. A. Y. Borisevich, A. R. Lupini, S. J. Pennycook, Depth sectioning with the aberration-corrected scanning transmission electron microscope. *Proc. Natl. Acad. Sci. U.S.A.* **103**, 3044–3048 (2006).

55. C. M. O'Leary, G. T. Martinez, E. Liberti, M. J. Humphry, A. I. Kirkland, P. D. Nellist, Contrast transfer and noise considerations in focused-probe electron ptychography. *Ultramicroscopy* **221**, 113189 (2021).

56. A. Strauch, D. Weber, A. Clausen, A. Lesnichaia, A. Bangun, B. März, F. J. Lyu, Q. Chen, A. Rosenauer, R. Dunin-Borkowski, K. Müller-Caspary, Live processing of momentum-resolved STEM data for first moment imaging and ptychography. *Microsc. Microanal.* **27**, 1078–1092 (2021).

57. P. M. Pelz, I. Johnson, C. Ophus, P. Ercius, M. C. Scott, Real-time interactive 4D-STEM phase-contrast imaging from electron event representation data: Less computation with the right representation. *IEEE Signal Process. Mag.* **39**, 25–31 (2022).

58. L. Liu, D. Zhang, Y. Zhu, Y. Han, Bulk and local structures of metal–organic frameworks unravelled by high-resolution electron microscopy. *Commun. Chem.* **3**, 99 (2020).

59. X. Li, I. Lazić, X. Huang, M. Wirix, L. Wang, Y. Deng, T. Niu, D. Wu, L. Yu, F. Sun, Imaging biological samples by integrated differential phase contrast (iDPC) STEM technique. *J. Struct. Biol.* **214**, 107837 (2022).
